# The Prevalence and Associated Risk Factors of Children With Reading Disabilities in a Multiethnic City: A Cross-Sectional Study

**DOI:** 10.3389/fped.2022.864175

**Published:** 2022-06-30

**Authors:** Yanan Feng, Qi Liu, Xinyan Xie, Qi Jiang, Kaiheng Zhu, Pei Xiao, Xiaoqian Wu, Pengxiang Zuo, Ranran Song

**Affiliations:** ^1^Department of Nursing, Shihezi University School of Medicine, Shihezi, China; ^2^MOE (Ministry of Education) Key Lab of Environment and Health, School of Public Health, Department of Maternal and Child Health, Tongji Medical College, Huazhong University of Science and Technology, Wuhan, China

**Keywords:** reading disabilities, Chinese Children, prevalence, risk factor, ethnic minorities

## Abstract

Numerous studies have been conducted to explore the risk factors for reading disabilities (RDs) among children. Based on these studies, factors such as gender, socioeconomic status, and the home literacy environment have been widely found to be associated with children who have RDs. However, children from a multiethnic city are seldom investigated. This study aimed to explore the prevalence of RDs and the potential environmental risk factors in Shihezi, Xinjiang, where people of multiple ethnicities, including Han and ethnic minority children, live and study together. A cross-sectional study was conducted in the city of Shihezi. A two-stage sampling strategy was applied to randomly select six primary schools in the city. In total, 6,539 students in grades two to six participated in this study. There were 6,065 valid questionnaires obtained for further analyses. We used the Dyslexia Checklist for Chinese Children and the Pupil Rating Scale to screen for the children with RDs. The χ^2^ test and multivariate logistic regression were employed to reveal the potential risk factors of RDs. The prevalence of children with RDs was 3.38% in Shihezi City and was significantly different between Han (3.28%) and Uighur (7.42%) children (*P* < 0.05). There was no significant difference in the prevalence of RDs between Han and Hui children. Among these children with RDs, the gender ratio of boys to girls was nearly 2:1. Multiple logistic regression analysis showed that gender (*P* < 0.01), learning habits (*P* < 0.01), and the home literacy environment (*P* < 0.01) were associated with RD. The results may be useful in the early identification and intervention of children with RDs, especially among ethnic minorities.

## Introduction

Reading disabilities (RDs) are a particular neurodevelopmental learning disorder, defined as a specific and significant impairment in reading ability that cannot be explained by deficits in intelligence, learning opportunities, motivation, or sensory acuity, also known as dyslexia. It can occur in areas of basic reading skills, written expression, listening, and speaking (1, 2). Children or adolescents with RDs may experience psychosocial consequences such as anxiety, depression, and low self-esteem (3–5), and they may have difficulties attaining educational degrees or high-income levels in their future life (6, 7).

Approximately 3–12% of individuals around the world have RDs (8). Studies conducted in English-speaking countries show a wide prevalence range of dyslexia (from 5 to 17.5%) (9–13). In China, the prevalence of dyslexia in school-aged children ranges from 3.0 to 12.6% (14). Epidemiological surveys have reported that the prevalence rates of dyslexia in Guangzhou, Xinjiang, Qianjiang, and Shantou are 5.4, 7.0, 3.9, and 5.4% (15–18), respectively.

Previous cross-sectional studies have revealed some of the risk factors associated with RDs, such as gender (19), socioeconomic status, parental educational levels, the home literacy environment (15), a difficult vaginal delivery, and preterm birth (9). Nevertheless, the risk factors associated with RDs could vary across different languages, races, and cultures. Identification of the risk factors for RDs may be helpful to increase understanding of the pathogenic mechanism.

Xinjiang is located in northwestern China and is a multiethnic settlement, where Central and Western Asian cultures converge. The region is populated by various ethnic groups, of which Uyghur and Han are the major ones. Shihezi City is located in the northern part of the Xinjiang Uygur Autonomous Region, China, which has a population of 624,400 and an area of 456.84 km^2^. In this city, there are 36,000 ethnic minorities, accounting for 5.4% of the population, and it is home to 27 ethnic groups, including the Han, Hui, Uighur, and Kazakh. Most ethnic minorities have their own unique genetic and cultural backgrounds, and they differ widely from European and Han people in terms of their beliefs, lifestyles, and languages. In contrast to that of Han children, the national languages of minority children, such as Uighur and Kazakh children, belong to the Altaic-Turkic language family, which is different from the Chinese language (belonging to the Sino-Tibetan language family). Children of ethnic minorities, on the one hand, have lived in a Chinese environment from birth and have learned unified Mandarin Chinese and standardized Chinese characters since kindergarten (approximately 3 years old). Chinese is widely used in their daily lives. On the other hand, ethnic minority parents often communicate with their children in their own language at home and teach their children to read and write their own traditional ethnic characters. Learning Chinese remains a particularly challenging task for children from ethnic minority groups, whose first language is not Chinese (20). Zhao et al. found that in Kashgar and Aksu, cities of Xinjiang Province, the rate of RDs in Uighur children is 7.93%, which is higher than the positive screening rate for Han children in the same region (3.89%) (16). Children from minority groups receive education in both their own language and Chinese from childhood, and the mechanism of RDs may be different from that in Han children (21).

This study aimed to investigate the prevalence of RDs in the multiethnic city and to explore if there exist differences in the prevalence of the RDs and potential risk factors between Han and ethnic minority children.

## Materials and Methods

### Study Design and Participants

This study was based on an ongoing program named Tongji Reading Environment and Dyslexia (READ) research. Our previous studies introduced this program (22).

A cross-sectional epidemiological study was designed for screening the children with RDs in Shihezi City. There are five districts and two towns in Shihezi. We randomly selected one primary school from each district and one primary school from each town. A total of six public schools were included in this study. All schools use Mandarin Chinese and standardized Chinese characters in their teaching activities and implement a mixed class establishment policy (mixed classes of Han and ethnic minorities). The Dyslexia Checklist of Chinese Children (DCCC) and the Pupil Rating Scale-Revised Screening for Learning Disabilities (PRS) was used to screen for Chinese RDs. The DCCC is a widely used, validated parent-reported scale designed to assess reading ability in students in grades two through six in China. The initial scale consists of 57 items, and the case definition of RDs was based on the Diagnostic and Statistical Manual of Mental Disorders (DSM-IV) and International Classification of Diseases (ICD-10) diagnostic criteria for learning disorders, as well as clinical symptoms described in the relevant references (23). Among these 57 items, 55 had loading on eight factors, including vocabulary comprehension deficit, visual deficit in word recognition, auditory deficit in word recognition, spelling deficit, written expression, attention deficit, oral language deficit, and poor reading habits. The score for each item ranged from 1 to 5 (1 = never, 2 = rarely, 3 = sometimes, 4 = often, and 5 = always), and a higher score indicated poor reading ability. The DCCC was completed by the child’s parent or guardian.

The PRS is a convenient tool for assessing learning ability in children and is widely used in China. It consists of 24 items that depict 5 components, namely, listening comprehension, time and spatial judgment, social behavior, motion ability, and memory and language ability. The head teacher was invited to complete the PRS scale depending on the students’ performance at school. The details of these two scales are available from our previous studies (23).

A child with RDs was recognized on the basis of the following criteria. (a) Their score on the DCCC was two standard deviations higher than the norm. (b) The PRS was lower than 65 points. (c) Their score on a Chinese language test was below the tenth percentile among all children in the same grade. (d) The child did not suffer from an intellectual disability, brain injury, visual or auditory disorder, epilepsy, or other psychiatric diseases.

### Data Collection

Before data collection, we obtained permission from all selected schools and informed consent from the students and their parents or guardians on behalf of participants who were invited to participate in the study.

This study was conducted between 1 March and 30 April 2021 by one researcher and three students with bachelor’s degrees who were acquainted with the manual of each scale and experienced in epidemiological surveys. After the investigators explained the purpose of the study, the DCCC and PRS scales were completed by the guardians and head teachers, respectively, depending on the written instructions of the scales. Besides the scales, we invited parents to complete a questionnaire containing four aspects, namely, general information, children’s reading habits, parents’ lifestyle during pregnancy, and the home literacy environment. In brief, general information included children’s age, gender, height and weight, parental education, occupation, and family’s economic status. Children’s reading habits included items such as scheduled time spent watching TV and surfing the Internet, scheduled reading time, and scheduled activity reading. Eleven items reflected the home literacy environment, such as the parents’ attitudes toward extracurricular books, the frequency of book buying for children, whether the parents told stories to their children from early childhood, whether the parents had a reading routine, and whether the parents bought the books favored by their children. We collectively referred to ethnic groups other than Han as ethnic minorities.

### Statistical Analysis

A descriptive analysis was conducted using the mean ± standard deviation (SD) for quantitative variables and frequencies for qualitative variables.

Differences in quantitative and qualitative variables between dyslexic and non-dyslexic children were examined using the *t*-test or χ^2^-test. The variables that were statistically significant in the *t*-test or χ^2^-test were included in multivariate logistic regression. All *P*-values were two-tailed with a significance level of 0.05, and all statistical analyses were carried out using SPSS 25.0.

### Ethics Statement

Written informed consents were obtained from all participants, and the next of kin, caretakers, or guardians on the behalf of the participants involved in this study. This study was also approved by the Ethical Committee of the Medical Association of Tongji Medical College, Huazhong University of Science and Technology.

## Results

### Participant Characteristics

In total, there were 6,539 students from grades two to six in the selected schools, and 6,228 students returned their questionnaires. There were 6,065 questionnaires completed for a response rate of 97.38%. The participant characteristics are shown in Table 1. Among 6,065 participants, 3,050 (50.3%) were boys, and 3,015 (49.7%) were girls. Their age ranged from 7 to 13 years, with a mean age of 10.3 years (*SD* = 1.31).

**TABLE 1:** Descriptive statistics of the participants.

Variables	All population	Han children	Ethnic minorities children^a^	χ^2^*^/^t*	*P*
			
	(*n* = 6,065)	(*n* = 5,434)	(*n* = 631)		
			
	*n* (%)	*n* (%)	*n* (%)		
**General information**					
**Gender**				10.390	0.001
Boy	3,050 (50.3)	2,771 (51.0)	279 (44.2)		
Girl	3,015 (49.7)	2,663 (49.0)	352 (55.8)		
Age (years), Mean ± SD	10.7 ± 1.6	10.7 ± 1.5	10.8 ± 1.6	–1.194	0.233
**Grade**				6.653	0.155
Grade two	933 (15.4)	818 (15.1)	115 (18.2)		
Grade three	1,157 (19.1)	1,046 (19.2)	111 (17.6)		
Grade four	1,317 (21.7)	1,186 (21.8)	131 (20.8)		
Grade five	1,323 (21.8)	1,176 (21.6)	147 (23.3)		
Grade six	1,335 (22.0)	1,208 (22.2)	127 (20.1)		
**Income of family per year (RMB)**				168.604	0.000
Less than 30,000 Yuan	812 (13.4)	633 (11.6)	179 (28.4)		
Between 30,000 and 49,000 Yuan	1,105 (18.2)	961 (17.7)	144 (22.8)		
Between 50,000 and 99,000 Yuan	1,868 (30.8)	1,715 (31.6)	153 (24.2)		
Between 100,000 and 199,000 Yuan	1,572 (25.9)	1,469 (27.0)	103 (16.3)		
Between 200,000 and 299,000 Yuan	441 (7.3)	406 (7.5)	35 (5.5)		
More than 300,000 Yuan	267 (4.4)	250 (4.6)	17 (2.7)		
**Occupation of father**				49.120	0.000
Professional technical staff or management staff	1,642 (27.1)	1,516 (27.9)	126 (20.0)		
Business and service staff	1,621 (26.7)	1,432 (26.4)	189 (30.0)		
Farming, forestry, fishery worker	431 (7.1)	360 (6.6)	71 (11.3)		
Production worker, transport worker and the related occupations	1,129 (18.6)	1,012 (18.6)	117 (18.5)		
Classification of inconvenience	993 (16.4)	908 (16.7)	85 (13.5)		
Jobless	249 (4.1)	206 (3.8)	43 (6.8)		
**Father’s education level**				71.884	0.000
Junior high school or below	2,065 (34.0)	1,755 (32.3)	310 (49.1)		
Senior high school or equivalency	1,477 (24.4)	1,357 (25.0)	120 (19.0)		
Junior college	1,162 (19.2)	1,075 (19.8)	87 (13.8)		
College diploma or above	1,361 (22.4)	1,247 (22.9)	114 (18.1)		
**Occupation of mother**				30.592	0.000
Professional technical staff or management staff	1,527 (25.2)	1,399 (25.7)	128 (20.3)		
Business and service staff	1,618 (26.7)	1,435 (26.4)	183 (29.0)		
Farming, forestry, fishery worker	320 (5.3)	273 (5.0)	47 (7.4)		
Production worker, transport worker, and the related occupations	607 (10.0)	558 (10.3)	49 (7.8)		
Classification of inconvenience	868 (14.3)	793 (14.6)	75 (11.9)		
Jobless	1,125 (18.5)	976 (18.0)	149 (23.6)		
**Mother**’**s education level**				49.115	0.000
Junior high school or below	2,118 (34.9)	1,821 (33.5)	297 (47.1)		
Senior high school or equivalency	1,396 (23.0)	1,261 (23.2)	135 (21.4)		
Junior college	1,156 (19.1)	1,065 (19.6)	91 (14.4)		
College diploma or above	1,395 (23.0)	1,287 (23.7)	108 (17.1)		

*^a^We collectively referred to Uighur, Hui, and others as ethnic minorities.*

### The Prevalence of Reading Disabilities in Shihezi City

Among a total of 6,065 participants, 205 suffered from RDs. The rate of RDs was 3.4% in primary schools in Shihezi City. There were 134 (65.4%) boys and 71 (34.6%) girls, which suggested that the gender ratio in RDs was 1.9:1 (boy:girl). Among the children with RDs, there were 178 (86.8%) children of the Han nationality, 19 (9.3%) children of the Uighur nationality, and 6 (2.9%) children of the Hui nationality.

As shown in Table 2, the individual prevalence rates of RDs in different schools were 4.1, 2.6, 4.2, 3.3, 3.1, and 2.9%. There were no significant differences in prevalence across different schools (*P* > 0.05).

**TABLE 2:** The prevalence rate of reading disabilities (RDs) in different schools.

School	RD	Normal	Rate (%)	χ^2^	*P*
				6.107	0.296
Shihezi no. 1 primary school^a^	64	1,505	4.1		
Shihezi no. 2 primary school^a^	33	1,224	2.6		
Shihezi no. 11 primary school^a^	21	482	4.2		
Shizongchang no. 1 primary school^a^	50	1,450	3.3		
Shihezi no. 16 middle school^b^	18	569	3.1		
Shihezi no. 19 middle school^b^	19	630	2.9		

*^a^Full-time primary school.*

*^b^Nine-year school.*

As shown in Table 3, the rates of RDs among children of Han, Uighur, Hui, and other ethnic minorities were 3.28, 7.42, 2.82, and 1.48%, respectively. There were significant differences in prevalence across different ethnicities (*P* < 0.05). The rate of RDs was significantly higher for Uighur children than for children of other ethnicities.

**TABLE 3:** The rate of RDs in different ethnicities^a^.

Ethnic	RD	Normal	Rate (%)	χ^2^	*P*
				16.597	0.005
Han	178	5,256	3.28		
Uighur	19	237	7.42		
Hui	6	207	2.82		
Other minorities ethnic*^b^*	2	133	1.48		

*^a^There was significant differences between Han and Uyghur children (χ^2^ = 12.575, P = 0.000) and Uyghur and Hui children (χ^2^ = 4.886, P = 0.027) in rates separately, and no significant difference between Han and Hui children (χ^2^ = 0.137, P = 0.711).*

*^b^Other minority ethnicities including Kazakh, Mongolian, and Tujia.*

### Factors Associated With Reading Disabilities

There was a significant difference between genders (*P* < 0.05). More boys (134, 65.4%) suffered from RDs than girls (71, 34.6%). The parents’ education level was significantly lower among children who suffered from RDs than among normal children (*P* < 0.05). There were fewer fathers who had attended college (19, 9.3%) or postgraduate studies (2, 1.0%) and fewer mothers who had received a college education (19, 9.3%) in the RD group (Supplementary Table 1).

### Learning Habits

There was a significant difference in hours of surfing the Internet between the two groups (*P* < 0.05). In the RD group, 10.2% of children used the Internet for more than 4 h at a time, which was a higher proportion than in the normal group. Hours spent watching TV were also longer among children with RDs than among normal children (*P* < 0.05). In the RD group, more children could not follow the scheduled time for watching TV and surfing the Internet (72, 35.1%), while more children had a scheduled time for watching TV and surfing the Internet (3,994, 68.2%) in the normal group. Compared with normal children, fewer RD children had active learning habits (31, 15.1%), and more RD children had difficulty completing their homework (52, 25.4%) (Supplementary Table 2).

### Home Literacy Environment

Factors in the home literacy environment were found to be associated with RDs. In the RD group, more children started to learn Chinese characters (30, 14.6%) and to read Chinese books (86, 42.0%) after 5 years of age. In the RD group, there was a lower rate of parental storytelling (33, 16.1%), encouraging the child to read extra-curricular books (126, 61.5%), and buying the books that the children were interested in (114, 55.6%). The proportion of parents who seldom read books was 44.4%, which was higher than that in the normal group (1,724, 29.4%). With regard to the children’ scheduled reading time, there was a large percentage of children without a fixed reading time in the RD group (98, 48.7%), whereas only 30.1% (1,761) of children had no fixed reading time in the normal group. Furthermore, fewer children (27, 13.2%) had more than 100 books in their homes in the RD group than in the normal group (1,437, 24.5%) (Supplementary Table 3).

### Multivariate Logistic Regression

There was a significant association between RDs and gender, parents’ education level, learning habits, and the home literacy environment (Supplementary Tables 1–3). To estimate the effect sizes of these possible risk factors, we conducted a multivariate logistic regression analysis. From Table 4, we found a significant difference between genders. The odds ratio (OR) in boys who suffered from RDs was 1.5 times as high as that in girls. We found that never learning actively and always having difficulty completing homework were negatively associated with RDs in children. Studying Chinese characters earlier was protective against RDs (less than 3 years old: OR = 0.5, 95% CI: 0.3–0.9; 3–5 years old: OR = 0.6, 95% CI: 0.4–0.9). A low frequency of visiting the library was also a risk factor for RDs (occasionally: OR = 2.7, 95% CI: 1.1–6.2).

**TABLE 4:** Multivariate logistic regression analysis of associated factors for RDs.

Subjects	Variables	OR (95%CI)	*P*
All subjects			
	**Gender**		
	Female	1	
	Male	1.5 (1.1–2.1)	0.007
	**Active learning**		
	Never	1	
	Yes	0.1 (0.1–0.2)	0.000
	Sometimes	0.4 (0.3–0.6)	0.000
	**Difficulty completing homework**		
	Always	1	
	No	0.1 (0.0–0.1)	0.000
	Sometimes	0.3 (0.2–0.4)	0.000
	**Age of learning Chinese characters**		
	Five years old and more	1	
	Less than 3 years old	0.5 (0.3–0.9)	0.012
	3–5 years old	0.6 (0.4–0.9)	0.032
	**Frequency of going to library**		
	Often	1	
	Occasionally	2.7 (1.1–6.2)	0.024
	Sometimes	2.1 (0.9–4.7)	0.078
Han nationality	**Gender**		
	Female	1	
	Male	1.5 (1.0–2.0)	0.026
	**Active learning**		
	Never	1	
	Yes	0.1 (0.1–0.2)	0.000
	Sometimes	0.4 (0.2–0.6)	0.000
	**Difficulty completing homework**		
	Always	1	
	No	0.1 (0.1–0.2)	0.000
	Sometimes	0.3 (0.2–0.4)	0.000
	**Age of learning Chinese characters**		
	Five years old and more	1	
	Less than 3 years old	0.5 (0.3–0.9)	0.015
	3–5 years old	0.6 (0.3–0.9)	0.022
	**Frequency of going to library**		
	Often	1	
	Occasionally	2.8 (1.1–7.3)	0.037
	Sometimes	2.1 (0.8–5.3)	0.120
Ethnic minorities	**Difficulty completing homework**		
	Always	1	
	No	0.1 (0.0–0.1)	0.000
	Sometimes	0.1 (0.1–0.5)	0.001
	**Age of reading Chinese book**		
	Five years old and more	1	
	Less than 3 years old	1.3 (0.1–16.8)	0.833
	3–5 years old	0.2 (0.1–0.6)	0.008

For minority children, completing homework without difficulty or only sometimes having difficulty had a positive effect on RDs (OR = 0.1, *P* < 0.01). Reading Chinese books at an early age (3–5 years old) had a positive effect on children with RDs (OR = 0.2, *P* = 0.008).

## Discussion

This study focused on the prevalence and risk factors for children with RDs in a multiethnic city. From this study, the main findings were as follows. The prevalence of RDs was 3.4% in Shihezi City, 3.28% in Han children, 7.42% in Uighur children, and 2.82% in Hui children. The prevalence of RDs differed between Han and ethnic minority children. Factors including learning habits and the home literacy environment were significantly associated with RDs.

Due to differences in the methods used to diagnose RDs and cultural differences, the prevalence of RDs was not consistent between studies. The prevalence of dyslexia in China was reported to range from 3.0 to 12.6% (14). In our study, the prevalence of RDs was 3.4%. According to the International Classification of Diseases (ICD-10), RDs are a specific and significant impairment in the development of reading skills that is not solely accounted for by mental age, visual acuity problems, or inadequate schooling (24). RDs are specific cognitive disorders, which cannot be explained by more general factors. It is the general consensus that the core problem in RDs is difficulty decoding text, and those who experience severe difficulties with decoding will usually experience associated problems of reading comprehension (25), which requires a written language assessment with a standardized test. We believe that standardized tests can be used to diagnose dyslexia with greater sensitivity and specificity. However, in China, due to the large population, the DCCC scale is more suitable for screening a large sample population and facilitates early detection. This scale includes assessments of decoding, word spelling, and writing. To accurately screen out children with RDs, we excluded children with intellectual disabilities, brain injuries, visual, and auditory disorders, epilepsy, and other psychiatric diseases that may cause difficulties in learning to read. We then used the DCCC scale to screen children with RDs in a multiethnic city and obtained similar conclusions to those in other regions.

Due to the differences in linguistic and genetic backgrounds between Han and ethnic minority children, the rate of RDs varies between studies. Zhao et al. (16) found that the dyslexia prevalence in the Han group (3.9%) was significantly lower than that of the Uighur group (7.0%), which was in close agreement with our study. In our study, the prevalence of RDs among Uighur children was 7.42%, which was significantly higher than that of Han children. However, there was no significant difference in the prevalence of RDs between Han and Hui children. There were several possible reasons for this result. The customs of the Hui nationalities are similar to those of the Han, Mongolian, and Uighur nationalities. Chinese is the general language of Hui nationalities, while the Uighurs have their own unique living habits and customs. Uighur is widely spoken in their family life. Uighur, an alphabetical language with a linear, one-dimensional alphabet (26), differs from Chinese (ideograph language) in linguistic characteristics (27). Most school-age Han and Hui children are Chinese monolinguals, whereas almost all school-age Uighur children are Uighur-Chinese bilinguals. They usually use their own language in communication with their families at home and use Mandarin Chinese at school and in social activities. Given these circumstances, children’s Chinese learning may be affected by Uighur learning habits (such as reading direction), and they easily get confused with the initials and rhymes. Previous studies have shown that when children are educated in two languages, their learning of the second language may be affected by the first language (28–30). Due to the differing characteristics of Uighur and Chinese, a bilingual imbalance has occurred in Uighur children. The reading direction of Uighur is from right to left, while that of Chinese is from left to right. Although Chinese is not considered a full second language for ethnic minority children, there are still some differences compared with Han children who were exposed to Chinese at birth. This makes it more difficult for them to learn Chinese. This shows that the bilingual environment may be one of the reasons why the prevalence of RDs among Uighur children is higher than that in other ethnic children. Further research in this area is warranted in the future. In addition to language differences, Uighurs rarely intermarry with other nationalities due to their cultural customs, so their population mobility, and genetic drift are low (31). The Uighur population has mixed ancestry and has been deemed to be genetically related to European and East Asian populations, so they may carry some genetic information, especially in terms of the molecular genetics of various diseases (32). Cultural, linguistic, and genetic differences may be responsible for the higher prevalence of RDs in Uighurs.

A large quantity of research also reported that more boys than girls suffer from dyslexia (12, 33). In this study, the gender ratio was nearly 2:1 in favor of boys among children with RDs. Logistic regression revealed that the risk for boys was 1.9 times as high as that in girls. Interestingly, we also found that the risk to boys was 1.5 times as high as that in girls among Han children, while there were no obvious gender differences among ethnic minorities. Previous studies found that the gender imbalance may be related to genetic differences, inherited features, and the development of cognition. One recent genetic study found that the CNTNAP2 gene variant was associated with gender differences among dyslexic children (34). Functional brain imaging studies have shown that men and women exhibit different brain activation patterns during speech processing (35). Neuroimaging and genetic studies have attributed this difference to the presence of hormone-related protective factors in women (36). Whether there are gender-based differences in the risk of RDs in ethnic minority children needs further research.

According to the results of logistic regression, we found that learning habits had a significant impact on the occurrence of dyslexia. Freeman et al. (37) found that active learning could improve students’ test scores. Gori et al. (38) observed that poorer temporal skills were correlated with lower reading skills in dyslexic children, suggesting that this temporal capability could be linked to reading abilities. Children with RDs usually have difficulty in reading, and failure to read reduces their motivation to learn, so children in this group have greater difficulty completing homework than those in the control group.

The home literacy environment was found to have an association with RDs in our study. The family literacy environment is an important predictor of children’s language and literacy development (39). Having an early learning environment in terms of vocabulary may lead to better literacy skills (40, 41). Early word-level literacy around 5.5 years had an effect on reading comprehension (42). Compared with dyslexic children, families of non-dyslexic children may provide a richer literacy environment (43). Learning to read in the early stages of education can lay the foundation for future literacy development and academic success (44–46). Starting to read Chinese books during kindergarten (3–5 years old) can reduce the risk of RDs in children of ethnic minorities. To make minority children more adaptable to the comprehensive Chinese teaching model, Xinjiang began to implement preschool bilingual education in 2005 to help minority children integrate into the Chinese language environment as early as possible. Our results proved it. It is recommended that parents of ethnic minorities should help their children be exposed to Chinese and overcome language barriers as early as possible. A good home literacy environment can increase children’ interest in reading and improve children’ reading skills (47). Based on our results, early literacy and reading more books can help to reduce the risk of RDs for both Han children and minority children.

This study showed that the learning habits and home literacy environment may be associated with RDs. The results of our study identified the risk factors that were associated with differences in the prevalence of RDs between Han and ethnic minority children. However, this study still has several limitations. This study was carried out in one city in China, and we could only generalize the prevalence to other cities that are similar to Shihezi City. Considering the large population in China, it is hard to screen the child with RD-based schools using the standardized tests that are conducted individually. The standardized tests may be considered to use for further research in the future.

Nevertheless, more studies involving a greater diversity of areas and races are urgently needed to prove the reliability and validity of DCCC in the future. There were many risk factors associated with RDs, and only some of these were analyzed in our study. In addition to the environmental factors, the etiology of RDs also involves neurology, genetics, and psychology. Further research is required to understand the genetic mechanisms underlying RDs in ethnic minority children and whether they differ from those in Han children.

## Data Availability Statement

The original contributions presented in this study are included in the article/Supplementary Material, further inquiries can be directed to the corresponding author/s.

## Ethics Statement

The studies involving human participants were reviewed and approved by written informed consents were obtained from all participants and the next of kin, caretakers, or guardians on the behalf of the participants involved in the study. Besides, the study was approved by the Ethical Committee of Medical Association of Tongji Medical College, Huazhong University of Science and Technology. Written informed consent to participate in this study was provided by the participants’ legal guardian/next of kin.

## Author Contributions

YF contributed to the conceptualization, methodology, formal analysis, investigation, and writing—original draft. QL, XX, QJ, KZ, PX, and XW collected the data and carried out the initial analysis. PZ and RS conceptualized and designed the study, collected the data, drafted the initial manuscript, reviewed, and revised the manuscript. All authors contributed to the article and approved the submitted version.

## Conflict of Interest

The authors declare that the research was conducted in the absence of any commercial or financial relationships that could be construed as a potential conflict of interest.

## Publisher’s Note

All claims expressed in this article are solely those of the authors and do not necessarily represent those of their affiliated organizations, or those of the publisher, the editors and the reviewers. Any product that may be evaluated in this article, or claim that may be made by its manufacturer, is not guaranteed or endorsed by the publisher.
