# Supplementary material for: The Prevalence and Associated Risk Factors of Children With Reading Disabilities in a Multiethnic City: A Cross-Sectional Study
Source: Front Pediatr. 2022 Jun 30;10:864175. doi: 10.3389/fped.2022.864175 (PMC9282866; doi:10.3389/fped.2022.864175)
Supplement: Supplementary file 1 [file Table_1.doc]

Supplementary Material

Supplementary Table 1. Analytical statistics for the RD and normal groups (General information).

|  | **All subjects** | |  |  | **Han children** | |  |  | **Ethnic minorities children ^a^** | |  |  |
| --- | --- | --- | --- | --- | --- | --- | --- | --- | --- | --- | --- | --- |
| **Variables** | **RD**  **(n = 205)** | **Normal**  **(n = 5860)** | ***χ^2^*** | ***P*** | **RD**  **(n = 178)** | **Normal**  **(n = 5256)** | ***χ^2^*** | ***P*** | **RD**  **(n = 27)** | **Normal**  **(n = 604)** | ***χ^2^*** | ***P*** |
|  | **n(%)** | **n(%)** |  |  | **n(%)** | **n(%)** |  |  | **n(%)** | **n(%)** |  |  |
| **Gender** |  |  | 19.293 | 0.000 |  |  | 13.647 | 0.000 |  |  | 7.823 | 0.005 |
| Male | 134(65.4) | 2916(49.8) |  |  | 115(64.6) | 2656(50.5) |  |  | 19(70.4) | 260(43.0) |  |  |
| Female | 71(34.6) | 2944(50.2) |  |  | 63(35.4) | 2600(49.5) |  |  | 8(29.6) | 344(57.0) |  |  |
| **Occupation of father** |  |  | 19.317 | 0.002 |  |  | 15.897 | 0.007 |  |  | 5.741 | 0.332 |
| Professional technical staff  or management staff | 3115.1) | 1611(27.5) |  |  | 29(16.3) | 1487(28.3) |  |  | 2(7.4) | 124(20.5) |  |  |
| Business and service staff | 68(33.2) | 1553(26.5) |  |  | 59(33.1) | 1373(26.1) |  |  | 9(33.3) | 180(29.8) |  |  |
| Farming, forestry, fishery worker | 13(6.3) | 418(7.1) |  |  | 10(5.6) | 350(6.7) |  |  | 3(11.1) | 68(11.3) |  |  |
| Production worker,  transport worker and the  related occupations | 43(21.0) | 1086(18.5) |  |  | 35(19.7) | 977(18.6) |  |  | 8(29.6) | 109(18.0) |  |  |
| Classification of inconvenience | 36(17.6) | 957(16.3) |  |  | 34(19.1) | 874(16.6) |  |  | 2(7.4) | 83(73.7) |  |  |
| Jobless | 14(6.8) | 235(4.0) |  |  | 11(6.2) | 195(3.7) |  |  | 3(11.1) | 40(6.6) |  |  |
| **Father’s education level** |  |  | 18.212 | 0.000 |  |  | 15.883 | 0.001 |  |  | 2.945 | 0.400 |
| Junior high school or below | 82(40.0) | 1983(33.8) |  |  | 69(38.8) | 1686(32.1) |  |  | 13(48.1) | 297(49.2) |  |  |
| Senior high school  or equivalency | 58(28.3) | 1419(24.2) |  |  | 51(28.7) | 1306(24.8) |  |  | 7(25.9) | 113(18.7) |  |  |
| Junior college | 44(21.5) | 1118(19.1) |  |  | 39(21.9) | 1036(19.7) |  |  | 5(18.5) | 82(13.6) |  |  |
| College diploma or above | 21(10.2) | 1340(22.9) |  |  | 19(10.7) | 1228(23.4) |  |  | 2(7.4) | 112(18.5) |  |  |
| **Occupation of mother** |  |  | 20.719 | 0.001 |  |  | 17.356 | 0.004 |  |  | 6.745 | 0.240 |
| Professional technical staff  or management staff | 28(13.7) | 1449(25.6) |  |  | 26(14.6) | 1373(26.1) |  |  | 2(7.4) | 126(20.9) |  |  |
| Business and service staff | 76(37.1) | 1542(26.3) |  |  | 65(36.5) | 1370(26.1) |  |  | 11(40.7) | 172(28.5) |  |  |
| Farming, forestry, fishery worker | 9(4.4) | 311(5.3) |  |  | 6(3.4) | 267(5.1) |  |  | 3(11.1) | 44(7.3) |  |  |
| Production worker,  transport worker and the  related occupations | 24(11.7) | 583(9.9) |  |  | 20(11.2) | 538(10.2) |  |  | 4(14.8) | 45(7.5) |  |  |
| Classification of inconvenience | 30(14.6) | 838(14.3) |  |  | 28(15.7) | 765(14.6) |  |  | 2(7.4) | 73(12.1) |  |  |
| Jobless | 38(18.5) | 1087(18.5) |  |  | 33(18.5) | 943(17.9) |  |  | 5(18.5) | 144(23.8) |  |  |
| **Mother’s education level** |  |  | 22.662 | 0.000 |  |  | 20.421 | 0.000 |  |  | 3.055 | 0.383 |
| Junior high school or below | 83(40.5) | 2035(34.7) |  |  | 71(39.9) | 1750(33.3) |  |  | 12(44.4) | 285(47.2) |  |  |
| Senior high school  or equivalency | 57(27.8) | 1339(22.8) |  |  | 50(28.1) | 1211(23.0) |  |  | 7(25.9) | 128(21.2) |  |  |
| Junior college | 46(22.4) | 1110(18.9) |  |  | 40(22.5) | 1025(19.5) |  |  | 6(22.2) | 85(14.1) |  |  |
| College diploma or above | 19(9.3) | 1376(23.5) |  |  | 17(9.6) | 1270(24.2) |  |  | 2(7.4) | 106(17.5) |  |  |

^a^ :We collectively refer to ethnic groups other than Han as ethnic minorities.
